# The macro-eco-evolutionary interplay between dispersal, competition and landscape structure in generating biodiversity

**DOI:** 10.1098/rstb.2023.0140

**Published:** 2024-08-12

**Authors:** O. Hagen, D. S. Viana, T. Wiegand, J. M. Chase, R. E. Onstein

**Affiliations:** ^1^ German Centre for Integrative Biodiversity Research (iDiv) Halle-Jena-Leipzig, Leipzig, Germany; ^2^ Department of Ecological Modelling, UFZ - Helmholtz Centre for Environmental Research, Leipzig, Germany; ^3^ Estación Biológica de Doñana, CSIC, Seville, Spain; ^4^ Naturalis Biodiversity Center, Leiden 2333 CR, Netherlands

**Keywords:** process-based models, intermediate dispersal, species interactions, speciation, biodiversity

## Abstract

Theory links dispersal and diversity, predicting the highest diversity at intermediate dispersal levels. However, the modulation of this relationship by macro-eco-evolutionary mechanisms and competition within a landscape is still elusive. We examine the interplay between dispersal, competition and landscape structure in shaping biodiversity over 5 million years in a dynamic archipelago landscape. We model allopatric speciation, temperature niche, dispersal, competition, trait evolution and trade-offs between competitive and dispersal traits. Depending on dispersal abilities and their interaction with landscape structure, our archipelago exhibits two ‘connectivity regimes’, that foster speciation events among the same group of islands. Peaks of diversity (i.e. alpha, gamma and phylogenetic), occurred at intermediate dispersal; while competition shifted diversity peaks towards higher dispersal values for each connectivity regime. This shift demonstrates how competition can boost allopatric speciation events through the evolution of thermal specialists, ultimately limiting geographical ranges. Even in a simple landscape, multiple intermediate dispersal diversity relationships emerged, all shaped similarly and according to dispersal and competition strength. Our findings remain valid as dispersal- and competitive-related traits evolve and trade-off; potentially leaving identifiable biodiversity signatures, particularly when trade-offs are imposed. Overall, we scrutinize the convoluted relationships between dispersal, species interactions and landscape structure on macro-eco-evolutionary processes, with lasting imprints on biodiversity.

This article is part of the theme issue ‘Diversity-dependence of dispersal: interspecific interactions determine spatial dynamics’.

## 1. Introduction

Understanding the mechanisms that drive dispersal as well as their effects on biodiversity is essential to predicting how ecosystems respond to environmental changes [1–4]. Although our mechanistic understanding of single-species dispersal dynamics has progressed rapidly over the last decade (e.g. [5]), the consideration of the interplay between species interactions and dispersal over ecological and evolutionary time scales is limited. Considering interactions between landscape structure, species dispersal is relevant to many issues in community ecology, invasion biology and conservation biology [6].

Dispersal, species interactions and landscape structure generate and erode biodiversity at multiple scales through multiple pathways [4,7–12]. Dispersal, for example, is linked to the isolation of populations, which impedes gene flow and promotes allopatric speciation [13–17]. While high gene flow impedes the accumulation of the genetic incompatibilities necessary to achieve reproductive isolation, low gene flow might prevent local adaptation [18] and can decrease the ability of species to track environmental variation, thereby potentially reducing biodiversity [19,20]. Species interactions can likewise impact speciation and extinction dynamics by affecting survival and population connectivity. For example, mutualistic interactions with frugivorous animals have shaped the dispersal and, hence, speciation rates of fleshy fruited plants [21]. Also, competition and facilitation can influence species geographical ranges [22], colonization [23] and survival [18]. Therefore, colonization and the isolation of populations is shaped by dispersal abilities, species interactions and landscape structure [24]. However, these eco-evolutionary processes are not independent of each other, and their interactions within a spatial and temporal context [25] can lead to complex patterns of biodiversity through space and time [26–28].

Empirical evidence points towards a hump-shaped relationship between species diversity across dispersal abilities [15,29,30]. Over evolutionary time, intermediate rates of dispersal can lead to the highest speciation rates and hence to maximum biodiversity [17]. Specifically, low dispersal can lead to low colonization and geographically restricted species, while high dispersal can lead to high species-wide genetic homogenization via gene flow. Thus, intermediate dispersal can represent middle ground for successful colonization while maintaining the isolation necessary for populations to achieve reproductive isolation followed by allopatric speciation, ultimately leading to the highest alpha (α) and gamma (γ) diversity. Similarly, intermediate dispersal should increase phylogenetic diversity (PD) because communities will be composed of a combination of phylogenetically distantly related colonizers and phylogenetically related species emerging from nearby speciation events. In contrast, increasing dispersal should decrease the turnover of species across assemblages, i.e. beta diversity (β), since it homogenizes community composition across space. Moreover, landscape structure (e.g. the degree of fragmentation and how environmental characteristics are distributed in space) can mediate dispersal among habitat patches and is crucial for reconciling contradictory findings regarding the relationship between dispersal and diversification [30].

Contrary to dispersal, increasing competition can decrease diversity through competitive exclusion monotonically. Competition controls speciation and extinction dynamics through density-dependent interactions, impacting species coexistence and geographical range [31]. As such, when there is no competition (i.e. no decrease in population size owing to the presence of other species), we would expect the highest α, γ and phylogenetic diversity overall. Beta diversity should increase with competition owing to higher local extinction rates and the differentiation of community composition as species adapt to or are limited by varying competitive pressures across different habitats [32]. For example, strong competition with other species can promote niche evolution such as the specialization towards specific environmental conditions [33–35]. This, in turn, alters species geographical ranges and the isolation of populations depending on the arrangement of the landscape that feeds back on dispersal-mediated macro-eco-evolutionary processes.

These eco-evolutionary processes are mediated by species traits (defined here as evolving and measurable local population average characteristics), which influence how species respond to changing environments and biotic conditions [10,36–39]. Traits related to climatic niches, dispersal and competition can directly and indirectly shape biodiversity [40–43]. For instance, thermal optimum and range, also referred to as thermal niche, limits the range of ecological conditions a species can tolerate (e.g. [5]), thus affecting population sizes, species distribution, and the ability of a species to successfully disperse, settle, and adapt to changing environments [28,37,43–46]. Moreover, empirical and theoretical studies suggest allometric, genetic and developmental constraints, often leading to correlations among traits, sometimes referred to as ‘trait syndromes’ [47–50]. One example, among many others, is the trade-off between dispersal and competition [31,51–53]. At ecological time scales, trade-offs between dispersal and competition may allow species to coexist [48,54]. These trade-offs impose physiological limits that minimize the chances for species to evolve into what may be thought of as a ‘super species’ that impedes coexistence [47,50,55].

Although we know that the interplay between dispersal, competition and landscape structure affects the evolution and maintenance of biodiversity, it remains unclear how and how much these eco-evolutionary feedbacks shape biodiversity over macroevolutionary time. Here, we investigate how dispersal (i.e. controlling colonization and the isolation of populations) and competition (i.e. decreasing population sizes owing to other species though density-dependent mechanisms), shape the emergence of various biodiversity patterns. We do so using a theoretical archipelago system, starting with only three species and few eco-evolutionary assumptions [56]. We compare biodiversity patterns (i.e. α, β, γ and PD) that emerge from three spatially explicit mechanistic models that differ in their underlying assumptions, directly or indirectly related to the ecology and evolution of dispersal and competition. The first model, M0, assumes fixed dispersal and competitive traits for all species within a simulation. The second model, ME, with E standing for evolution, allows dispersal and competitive traits to evolve and diverge with time between isolated populations. The third model, MET, with T standing for trade-off, constrains dispersal- and competitive-related traits. Using these three eco-evolutionary models (i.e. M0, ME and MET), we explore the full factorial combination of a large range of dispersal and competitive parameters to shed light on how biodiversity is conditional on the interaction between dispersal, competition and landscape structure.

## 2. Methods

**Figure 1.**
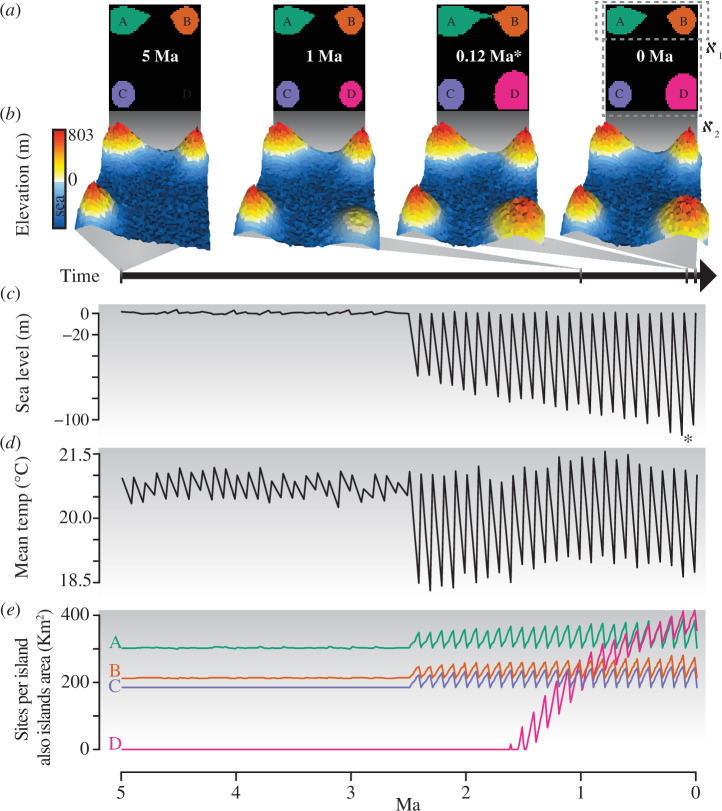
Our landscape is a theoretical archipelago system where site temperature (i.e. min and max temperature) and structure are generated by approximating topography, uplift dynamics and lapse rate, as well as global temperatures and sea level changes for the last 5 million years. (*a*) Four snapshots of the landscapes between 5 and 0 million years ago (Ma), starting at the first and ending at the final time step. Note the appearance of island D at ~1.5 Ma and the consequences of the lowest sea level point (*) to the general increase in island size and the disproportional distances between islands A and B. The dotted grey line at the final time step shows two connectivity regimes between islands (i.e. ϰ_1_ and ϰ_2_ connectivity regimes relative to dispersal abilities ranging from 0.15–0.55 to 0.55–1, respectively). (*b*) Four snapshots of dynamic topography (i.e. lowest and highest elevation across all times according to sea level at 0 Ma were respectively −115 m and 803 m), (*c*) sea level changes (relative to 0 Ma); and (*d*) temperature changes according to a lapse rate, here clumped as the mean temperature of all suitable sites; resulted in (*e*) dynamic number of sites per island corresponding to island area (km^2^).

**Figure 2.**
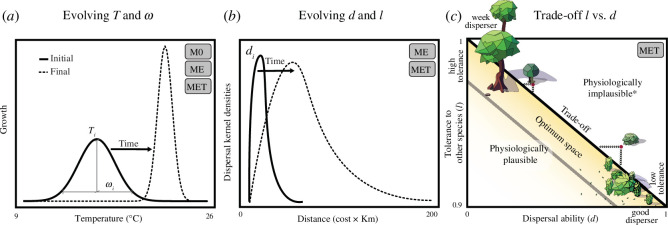
Eco-evolutionary models (M0, ME and MET) within three main assumptions (*a–c*). Note that all modes have an evolving thermal optimum $T_{i}$ and thermal range $\omega_{i}$ . ME and MET had evolving dispersal ( d ) and competitive ( l ) traits, while M0 had no evolving d or l and thus, all species within a simulation had equal d and l . MET assumes a trade-off between d and l . (*a*) Population growth changes according to site conditions (i.e. minimum and maximum temperature [9–26℃]) and evolving species thermal optimum T and thermal range ω through a Gaussian environmental function that considers a geometric mean of minimum and maximum local temperature. (*b*) Frequencies of dispersal events change according to a Weibull function that has scale changed by d resulting in concentrated short-range dispersal events for small d with increasingly larger and longer tails for larger d . (*c*) MET is the only case where d and l evolution and trade-offs are present, e.g. as postulated particularly for plants. A species at the top-left of the assumed trait trade-off optima is a good competitor, i.e. not affected by the population size of other species, but has a low dispersal trait and represents, for example, climax species that invest many resources in few propagules. A population at the bottom-right has low heterospecific tolerance but has high dispersal abilities. Imagine, for example, species that are short and produce many seeds but are quickly over shaded by species that on the other hand cannot produce so many propagules. Species that are at the assumed physiological implausibility space (red dot) are pushed back to the trade-off surface (arrows) randomly to the closest possible *d* and *l* combinations. Note that our model does not account for dispersal by other groups such as zoochoric plants, which could break the rules by which colonization success might depend on less dispersal energy allocation.

We simulated emergent biodiversity patterns in a dynamic theoretical archipelago over 5 million years (figure 1) using three alternative models (figure 2). We used empirically feasible elevation, temperature and sea level changes in our spatially explicit and dynamic landscape to stipulate the environmental suitability of sites (i.e. sites above the sea level at each respective time), minimum and maximum temperatures (i.e. $T_{min}$ , $T_{max}$ ) and the distances between sites and islands. Populations are characterized by the following traits: thermal optimum ( $T_{i}$ ), thermal range ( $\omega_{i}$ ), dispersal ability ( $d_{i}$ ) and tolerance to other species ( $l_{i}$ , a proxy for heterospecific competition strength). For each simulation, we kept track of trait variation, population size, occupation, species composition and overall biodiversity (α, β, γ and PD), as well as speciation and extinction events through time.

For each time step, we simulated speciation, dispersal, trait evolution and ecological dynamics of populations inhabiting different sites using gen3sis v.1.5.2, an engine for mechanistic eco-evolutionary biodiversity modelling [56]. First, geographically isolated populations (i.e. population clusters) accumulate genetic differentiation and are split into new species if genetic incompatibility is larger than a speciation threshold [13,14,57]. Second, dispersal allows the colonization of new sites and defines the population clusters (i.e. populations occupying sites that are reachable by dispersal). Third, we homogenize population traits within population clusters and update trait values randomly from a normally distributed trait evolution (i.e. non-directional change). Fourth, we determine population sizes of prior occupants and recent immigrants in each site according to the outcome of the species’ thermal niche (as defined by the population traits $T_{i}$ and $\omega_{i}$ and the site temperature, $T_{min}$ and $T_{max}$ ) and competition (assuming a fixed conspecific interaction and varying heterospecific competition).

### (a) Landscape

We approximate 5 million years of environmental dynamics (i.e. changes in topography, temperature and sea level) in a theoretical, but realistic, archipelago system. Sites (1×1 km^2^) form islands (~250 above sea level; i.e. potentially suitable sites) and have a minimum and a maximum temperature (i.e. $T_{min}$ and $T_{max}$ ) depending on site elevation and average global temperatures, which change every 10 000 years (i.e. after 501-time steps). According to approximations of global temperature and sea level changes [58,59] that intensified during the quaternary (~last 2.6 Ma), we oscillated temperatures periodically in a frequency of 100 ky (i.e. each 10th-time step) [60,61]. We set sites $T_{min}$ and $T_{max}$ according to a lapse-rate (i.e. a decrease in mean temperature with elevation). Overall minimum temperatures were 9℃ and maximum temperatures were 26℃. Differences between $T_{min}$ and $T_{max}$ ranged between zero and 5℃. Moreover, landscape structure defined geographical distance and resistance cost (also known as landscape permeability) accounting for site suitability and elevational differences [62–64]. Landscape resistance to dispersal was ‘four’ for unsuitable sites (i.e. ocean matrix) and ‘one’ for suitable sites with the addition of 0.1 per 100 m slope difference between source and destination site.

At 5 Ma, our archipelago had three islands (i.e. A, B and C) with topography unaltered over time, and one island (i.e. D) that started to appear around 1.5 Ma and increased its area over time. All four islands had central points equally spaced to each other. Islands A and B had their areas and proximity periodically increased as a result of a shallow land bridge, which decreased distance between islands A and B when sea levels were low. In contrast, islands C and D remained isolated through time (figure 1, electronic supplementary material, animation S1). Depending on the set dispersal traits, our landscape configuration created two connectivity regimes that could simultaneously enable colonization and isolation of populations between islands A and B (i.e. connectivity regime ϰ_1_) and between all islands (i.e. connectivity regime ϰ_2_), whereas connectivity regime ϰ_0_ did not allow for speciation events between islands.

### (b) Eco-evolutionary models

#### (i) Speciation

Speciation occurs through allopatry, which is dictated by changes in species ranges and the isolation of populations. Connected populations (also referred to as population clusters) result from species ranges, landscape structure and dispersal ability. Isolated populations (i.e. populations in different clusters) gradually diverge in their genetic incompatibility pairwise distance (i.e. by a +1 increment per time step) approximating a genetic drift for non-adaptive traits [13,14,16]. In contrast, connected populations (i.e. populations within clusters) decrease in their pairwise genetic incompatibility distances (i.e. by −1 per time step), gradually converging towards zero when connectivity between populations is maintained over several time steps. Speciation happens once the pairwise genetic incompatibility distance reaches a speciation threshold (s), representing the dynamic process of species formation through reproductive isolation without accounting for ecological speciation [65]. We used s = 65 (i.e. 650 kyrs), within the reported allopatric speciation rates [66–68]. Additionally, we varied s = [20–75] (i.e. 200–750 kyrs) in our sensitivity analysis to evaluate the effect of speciation thresholds on model outcomes.

#### (ii) Dispersal

Dispersal determines (i) the arrival of populations to new sites and (ii) the isolation of populations, both of which depend on population ranges, landscape structure and a dispersal kernel [7]. Dispersal distances are randomly drawn from a Weibull probability distribution ( φ,ψ ) with a shape parameter φ=2 and a scale parameter ψ ranging from 1 to 50, which is controlled by the dispersal trait ( d ). We account for landscape resistance when determining successful dispersal events, but environmental quality and species interactions determine successful colonization (settlement) and the formation of population clusters, since the latter depends on the ecological model component [56]. Arrivals are successful (arriving population size $N_{i}=0.05$ ) if dispersal events are larger than the minimal landscape resistance cost between pairs of occupied versus unoccupied sites (i.e. suitable sites that are uninhabited by a given species). Population clusters are formed similarly, but events are compared between pairs of occupied sites for a given species. For computational efficiency, for each time step and for a given species, the dispersal trait ( d ) is the population-size weighted dispersal of all populations ( P ) for a given species. Note that colonization events are only successful if arriving populations have a positive size N_f_ after the ecological processes have been considered at the end of each time step.

#### (iii) Trait evolution

The traits defining the thermal niche, dispersal and heterospecific competition, can have a maximum value of one, such that: T=1 is the highest thermal optimum (i.e. adaptation to 26℃ , the warmest available temperature at all times); ω=1 is the highest thermal range (i.e. populations can grow across all sites); d=1 is the highest dispersal (i.e. all sites can be colonized and gene flow is maintained across populations of the same species in all islands); and l=1 is the highest tolerance to other species and thus no heterospecific competition (i.e. population size is not reduced owing to competition with other co-occurring species $\alpha_{fh}=0$ ). After estimating the ecological equilibria, all connected populations receive the population-size-weighted average as the new trait values for the following time step. At each time step, traits change according to a normal distribution with mean zero and standard deviation (s.d.) of 0.001. $\alpha_{fh}$ dependent on l ranged from [0–0.1] (i.e. no competition and high heterospecific competition), and s.d. = 0.01, whereas all other traits ranged from [0–1]. Although our model focuses on allopatric speciation, trait divergence can result in isolation by spatial clustering and consequently speciation. In a smaller set of simulations, we considered that connected populations from the same species could differ in their average trait values by pulling population traits towards half of their distance to the weighted population average. This did not affect our results, but avoided speciation within islands for the special case d = 0. (see electronic supplementary material).

#### (iv) Ecology

Abiotic and biotic conditions determine species population sizes at each site, measured in hundreds of (implicit) individuals per km^2^. The local population size *N*_f_ *,* of a focal species *f* is determined by the species’ response to temperature (niche response) and the effect of con- and heterospecific competition derived from a Lotka–Volterra [69,70] interaction model [71].


(2.1) $$\frac{dN_{f}}{dt}\frac{1}{N_{f}}=r_{f}-\alpha_{ff}N_{f}-\alpha_{fh}\sum_{k\neq f}N_{k}$$

where *r*_f_ is the maximum per capita growth rate of a population of the focal species *f* during timesteps *dt*, that are much shorter than the overall simulation timesteps. The maximum per capita growth rate *r*_f_ depends on the site conditions by


(2.2) $$r_{f}=g\times \sqrt{n_{f}\left(T_{min}\right)\times n_{f}\left(T_{max}\right)}$$

where *g* is a species-independent constant parameter (i.e. *g = 0.1*) that gives the maximal value of $r_{f}$ across all sites, and $n_{f}\left(T\right)$ is a function that relates the maximum per-capita growth rate of population *f* at a given environmental temperature ( $T_{env}$ ), according to a Gaussian function (figure 2*a*) that considers $T_{env}$ at the site and two trait values: a thermal optimum (*T*_f_) and a thermal range ( $\omega_{f}$ ). Thermal range also determines the height of the Gaussian function given by $\frac{1}{\omega_{f}}$ and leads to a maximum per capita growth rate at the thermal optimum *T*_f_ of the focal species.


(2.3) $$n_{f}\left(T_{env}\right)=\frac{1}{\omega_{f}}\times exp\left(\frac{-{\left(T_{env}-T_{f}\right)}^{2}}{2\omega_{f}^{2}}\right)$$

Equation (2.2) assumes that maximum growth $r_{f}$ at a given site is proportional to the geometric mean of the focal population growth at $T_{min}$ and $T_{max}$ at a given site, which reflects how well the population can grow under the given local conditions. If the thermal range ( $\omega_{f}$ ) is very narrow (i.e. $\omega_{f}\to 0$ ), the species can only exist at sites with temperatures very close to the thermal optimum. If the thermal range is wide (i.e. $\omega_{f}\to 1$ ), the population can exist at a wide range of temperatures, but only at suboptimal values of $r_{f}$ since the height of the Gaussian curve declines with larger values of $\omega_{f}$ .

The relative population growth rate of the focal species f at the given site then decreases linearly with its own population size, depending on the conspecific interaction coefficient $\alpha_{ff}$ and the heterospecific interaction coefficient $\alpha_{fh}$ , which is the same for all heterospecifics (equation (2.1)). The heterospecific interaction coefficient ( $\alpha_{fh}$ ), here $\alpha_{fh}=1-l$ , describes the strength of competition of other species on the focal species. Thus, there is little effect of heterospecifics (i.e. $\sum_{k\neq f}N_{k}$ equation (2.1)) on a focal population when the interaction coefficient $\alpha_{fh}$ is low, i.e. the tolerance to other species is high ( l→1 ) and there is no heterospecific competition when l = 1.

We calculated the population sizes of all species for all sites, assuming ecological equilibria for every 10 000 years. We solved interactions at shorter timesteps dt (i.e. here 5 years), through the implementation of an analytical solution for the equilibrium of equation (2.1) [71]. The maximum per capita growth rate $r_{f}$ is the probability that one adult individual produces during the 5-year timesteps one offspring, and the interaction coefficients $\alpha_{ff}$ and $\alpha_{fh}$ reduce this probability per conspecific and heterospecific (implicit) individuals, respectively. In order to obtain equilibria under any parameter combination, the conspecific interaction coefficient α_ff_ was assumed to be the same for all species (i.e. α_ff_ = 0.2), whereas the heterospecific interaction coefficient α_fh_ varied among simulations and populations for models considering trait evolution. The heterospecific interaction coefficient ( $\alpha_{fh}$ ) describes diffuse competition at the population scale (i.e. the competitive effect of many neighbours of many species at the individual scale averages out). [71] showed that diffuse competition emerges in species-rich plant communities at the population scale from local crowding competition at the individual scale, even if the pairwise individual-level species interaction coefficients differ among different heterospecific species. We summarize the estimation of the equilibrium in the electronic supplementary material.

### (c) Simulation experiment

All models were initiated 5 Ma with three species, each with populations spread over all suitable sites on each island: species 1, 2 and 3 on islands A, B and C, respectively (figure 1). Initial populations had arrival sizes ( N=0.05 ), intermediate thermal ranges ( ω=0.4 ) and thermal optimum equal to the mean temperature at each site (figure 2*a*). Our full factorial exploration created combinations of simulations across dispersal kernels ( ψ ) ranging [1–50], regulated through the dispersal trait ( d ) and heterospecific interaction coefficients ( $\alpha_{fh}$ ) ranging [0–0.1], regulated through the competition trait ( l ). This systematic exploration of alternative scenarios allowed us to assess the impact of specific model parameters on the resulting biodiversity patterns given our dynamic landscape.

We ran 2000 simulations of each model M0, ME and MET. Our models had different assumptions related to trait evolution and trade-offs (figure 2). In the M0 model, we assumed no evolution of traits d or l (i.e. proxies for ψ and $\alpha_{fh}$ ). Thus, M0 serves as a reference on how dispersal and competition along with landscape structure affect biodiversity dynamics without the variability of ψ and $\alpha_{fh}$ for populations/species within a simulation. In the ME model, we allowed d and l to evolve freely without any constraints (figure 2*b*) within the same parameter ranges as in M0. This is realistic, as dispersal and competitive traits evolve over macroevolutionary time and across lineages. In the MET model, we imposed a trade-off on d and l constraining the parameters ψ and $\alpha_{fh}$ (figure 2*c*). The motivation behind the trade-off stems from empirical evidence [50], suggesting that species are rarely good competitors as well as good dispersers. We ran 2000 simulations for each of the three models with simulations covering unique starting d and l value combinations (full factorial). For MET, d and l staring value combinations that fell within a physiologically implausible space, were reallocated to the trade-off surface (figure 2*c*, electronic supplementary material, figure S3c). In total, we ran ~ 12 000 simulations including the ones used at the sensitivity analysis.

### (d) Analysis

For each simulation, we measured diversity, including taxonomic and phylogenetic diversity, using classical ecological and evolutionary metrics at the site, island and system levels (respectively local, patch and regional scales) [72–74]. Gamma diversity (γ) was the final regional taxonomic richness (number of species alive at the last time step), beta diversity (β%) was the proportional [75] Whittaker’s species turnover across all islands, i.e. 1 − mean(α/γ) [76], and mean αlpha diversity ( $\bar{\alpha }$ ) was the mean final local taxonomic richness per site. Mean phylogenetic diversity ( $\bar{PD}$ ) was the per-site average of the total community phylogenetic branch length [73,77]. Speciation and extinction events were tracked in space and time and are reported as proportions of events over the entire simulation. Proportions of islands involved in speciation events (speciation island prop.) were measured for each island, allowing a direct link of macro-evo-evolutionary processes with the landscape structure. Mean regional occupancy $\left(\bar{occup}\right)$ was calculated in km^2^ according to the mean number of sites occupied by all species through time. For further diversity metrics, see electronic supplementary materials.

## 3. Results

### (a) Multiple hump-shaped dispersal-diversity curves appeared according to landscape structure

**Figure 3.**
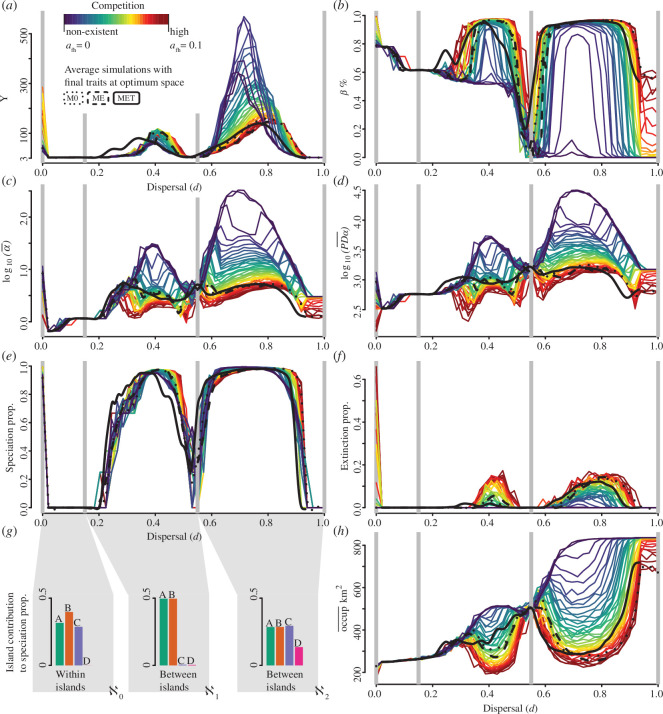
Summary statistics (y-axis), along a gradient (x-axis) of simulated initial dispersal trait ( d ). Coloured lines are only for M0 values of competition, measured as the heterospecific interaction coefficient ( $\alpha_{fh}$ ) and controlled by the tolerance to other species traits ( l ). Low competition $\alpha_{fh}\to 0$ is indicated by colder blue colours, while higher competition $\alpha_{fh}\to 0.1$ is indicated by warmer red colours. Smooth lines of M0, ME and MET simulations (respectively, dotted, dashed and solid black lines) with final mean trait inside the theoretical trait trade-off optimum space (figure 2*c*). M0 is used as a reference since dispersal and competition traits (i.e. proxies for ψ and $\alpha_{fh}$ ) are equal for all populations/species through time and within a simulation. Three connectivity regimes at dispersal values of d =0–0.15 (ϰ_0_); d =0.15–0.55 (ϰ_1_); and d =0.55–1 (ϰ_2_) are marked by grey vertical lines. Plots illustrate average values across the entire landscape at the final time step, based on species alive. (*a*) Gamma diversity γ. (*b*) Proportional beta Whittaker’s species turnover β. (*c*) Log 10 mean local alpha taxonomic diversity α. (*d*) Log 10 mean local phylogenetic diversity (i.e. local average of total community phylogenetic branch length). (*e*) Proportion of speciation events in a simulation over final γ diversity. (*f*) Proportion of extinction events in a simulation over final γ diversity. (*g*) Proportion of islands involved in speciation events for simulations within each connectivity regime. Speciation events are divided within and between islands, respectively, if speciation happens within (ϰ_0_) or between (ϰ_1_ and ϰ_2_) different islands. (*h*) Mean regional occupancy $\left(\bar{occup}\right)$ in km^2^, i.e. the mean number of sites occupied by all species through time.

The effect of dispersal on biodiversity through allopatric speciation was strongly determined by the landscape structure that shaped the connectivity regimes. Increases in dispersal ability led to a general increase in α, γ and phylogenetic diversity (figure 3*a,c,d*) and a decrease in β diversity (figure 3*b*) as a result of dispersal-mediated community homogenization (electronic supplementary material, figures S7*b*, S10, S21*b*, S24, S32*b*, S35 and S42*d*–f,). Species with dispersal trait d = [0.15–0.55] (i.e. connectivity regime ϰ_1_) were able to colonize closely connected islands (i.e. islands A and B) while maintaining populations isolated over long periods of time, leading to speciation (figure 3*e*), while species with dispersal trait d = [0.55–1] (i.e. connectivity regime ϰ_2_) were able to colonize all islands (i.e. islands A, B, C and D) while possibly also maintaining population isolation leading to speciation. Importantly, we found ‘intermediate dispersal’ regimes within each of the two connectivity regimes ϰ_1_ and ϰ_2_ which led to the highest α, γ and phylogenetic diversity (figure 3*a,c,d*) through increased allopatric speciation (figure 1, figure 3*e,g*). Increases in dispersal led to overall decreases in β-diversity, especially for low competition simulations (figure 3*b*). Finally, when dispersal approached zero, species could only colonize close-by sites, but not new islands. Extinctions were considerably higher for d ≈ 0 because species were unable to track their niche, thus suffering from environmental changes, such as sea level and temperature changes in the Quaternary (figure 3*f,h*, electronic supplementary material, figures S17, S19 and S30). Concordantly, all speciation events with d =[0–0.15] (i.e. connectivity regime ϰ0)—only observed at M0 with full trait homogenization—happened within islands, while between island regimes (ϰ_1_ and ϰ_2_) led to repeated allopatric speciation events as a result of successful colonization of nearby islands followed by repeated interrupted gene flow—observed in all models.

### (b) Competition with other species shifts diversity peaks towards higher dispersal abilities

Lower levels of heterospecific competition (i.e. higher heterospecific tolerance) generally led to higher α, γ and phylogenetic diversity (blue colours in figure 3*a,c,d*). Nevertheless, $\alpha_{fh}$ values did not impact diversity similarly throughout the dispersal gradient. Instead, they impacted diversity similarly within the two connectivity regimes ϰ_1_ and ϰ_2_ (see coloured lines for M0 in figure 3*a–f,h*). Specifically, low heterospecific competition led to higher γ diversity when the dispersal value was low within the connectivity regimes (i.e. *d* = 0.2–0.4 and *d* = 0.55–0.8; figure 3*a*). In contrast, while γ diversity for low dispersal (within a given connectivity regime) shows that lower competition leads to higher diversity, increasing dispersal reversed this relationship, i.e. higher dispersal under high competition led to higher diversity (within a given connectivity regime) (figure 3). This suggests that when dispersal is not limited, successful colonization events of distant communities may be rare but possible, allowing colonizers to avoid niche overlap and competitive pressure from heterospecifics, thereby increasing γ (but not α or PD) diversity (figure 3*a,c,d*, electronic supplementary material, figure S32). In other words, low competition only leads to higher γ diversity when dispersal is limited (i.e. when on the first half of the connectivity regimes ϰ_1_ and ϰ_2_, respectively around d = 0.2–0.4 and d = 0.55–0.8) (figure 3*a*). Moreover, high heterospecific competition led to the highest β diversity, contrasting with the prominent decrease in β diversity along the dispersal gradient in the absence of competition (figure 3*b*).

### (c) The effect of trait evolution and trade-offs on biodiversity

Adding unconstrained evolution (ME) and a trade-off (MET) to M0 resulted in overall lower α and γ diversity (see dotted, dashed and solid black lines for models M0, ME and MET in figure 3). Moreover, ME and MET removed most of the variability on biodiversity metrics attributed to competition across the dispersal gradient, most noticeably for β diversity on MET (electronic supplementary material, figure S42). Overall, models ME and MET showed similar final patterns to that of model M0, with an expected stronger selection of competition traits than on dispersal (electronic supplementary material, figures S43–S45, S50). MET demonstrated the most robust positive correlation between γ diversity and competition, as evidenced by its substantial role in increasing speciation rates alongside rising extinction events (electronic supplementary material, figures S44 and S48). Specifically, MET had the highest speciation enhancement owing to increased extinctions. The linear regression slopes between speciation and extinction proportions were 0.21 (95%CI: 0.18–0.24; *R*² = 0.18) for M0; 0.36 (95%CI: 0.32–0.41*; R*² = 0.22) for ME; and 0.48 (95%CI: 0.45–0.52; *R*² = 0.45) for MET, indicating a significant and, respectively, increasingly stronger positive trends. The inclusion of dispersal trait evolution constraints in MET altered both the magnitude and direction of dispersal trait changes (electronic supplementary material, figures S43 and S45), particularly under varying environmental stability conditions. Specifically, during 2.5–0 Ma, i.e. the dynamic period, there was a positive shift in all models while during 4.5–2.5 Ma, i.e. the stable period, the mean slope for dispersal evolution was negative, indicating a selection against dispersal in stable times. The slopes and *R*² values for the dispersal evolution through time across different periods were as follows: (M0) during the stable (slope = 1.10 × 10^−20^, *R*² = 0.505) and dynamic (slope = 8.09 × 10^−20^, *R*² of 0.495) period; (ME) during stable (slope = 1.87 × 10^−06^, *R*² = 0.441) and dynamic (slope = 4.64 × 10^−06^, *R*² = 0.443) period; (MET) during stable (slope = −5.14 × 10^−05^, *R*² = 0.612) and dynamic (slope = −6.36 × 10^−05^, *R*² = 0.628) period.

These findings suggest possible identifiable historical signatures of trait evolution, highlighting how species dispersal abilities have been shaped over time in response to environmental stability and dynamism. These signatures appear more strongly in simulations assuming a trade-off (i.e. MET), with diversity peaks generally moving towards smaller dispersal distances than in the ME model (figure 3, electronic supplementary material, figure S42). Multiple diversity peaks, especially visible at ϰ_1_ in MET (black lines in figure 3), are evident throughout the dispersal gradient, while species in ME simulations escaped competitive pressure by simply evolving an unconstrained tolerance to other species (electronic supplementary material, figure S45 *g*–*i*). Additionally, only in M0 and when assuming a full homogenization of traits per population cluster, α and γ diversity showed a peak under very low dispersal (i.e. d → 0). Moreover, extinctions at very low dispersal directly relate to the incapability of species to track their environmental niches in periods of environmental change (figure 3*f,h* and electronic supplementary material, figure S17). Once dispersal ability evolved (models ME and MET) or for M0 with less trait homogenization considered, speciation within islands was not observed. In these cases, species either (i) evolved a slightly higher dispersal ability avoiding geographical isolation within islands or (ii) in the M0 case with less trait homogenization, species had larger geographical ranges because population variability allowed for local adaptations, consequently maintaining population connectivity.

## 4. Discussion

We employed an integrative theoretical framework combining principles of dispersal ecology and evolution, supported by spatially explicit models, to explore the complex interplay between dispersal, competition and landscape structure on biodiversity [26,78]. Our findings challenge the notion of additive effects between these factors and biodiversity, revealing multiple diversity peaks across a dispersal gradient in a relatively simple landscape. Moreover, these diversity peaks change in accordance with competitive strength across all our eco-evolutionary models.

First, we show that an intermediate dispersal within connectivity regimes in a landscape leads to the highest speciation rates, and hence the highest α, γ and phylogenetic diversity. The emerging diversity peaks reflect the balance between colonization success and maintenance of reproductive isolation until speciation, resulting in diversity peaks for a given connectivity regime. The importance of landscape structure in generating biodiversity is evident at the absolute intermediate dispersal along our gradient (*d* = 0.55) that shows a drop in overall diversity (figure 3). At this dispersal ability, all sites within islands A and B are connected (shown by speciation drop-in figure 3*e*), yet colonization of islands C and D is not possible (shown by saturation of no competition line in figure 3*h*), thereby hindering the process of allopatric speciation. This emphasizes that an ‘absolute intermediate dispersal’ *per se* does not lead to the highest overall biodiversity. Instead, the emergence of biodiversity is strongly context-dependent, with dispersal ability and landscape structure interactively shaping connectivity regimes and speciation.

Second, we show that the interaction between dispersal and landscape structure is significantly influenced by heterospecific competition. Low competition generally enhances α, γ and phylogenetic diversity (blue colours in figure 3*a,c,d*), in line with the expectation that more species can coexist if competition is low. However, at higher dispersal abilities within connectivity regimes, increased competition paradoxically promotes higher γ-diversity, but not α-diversity (red colours in figure 3*a,b,c*). This may be attributed to good dispersers being able to successfully colonize remote communities and establish in these, while remaining isolated from conspecifics owing to competition-driven extinction in surrounding communities, thus leading to speciation. Competition selected for temperature specialists, leading to lower occupancy (figure 3*h*, electronic supplementary material, figure S45*a*–*c*) and consequently more isolated local populations. This promoted speciation at, relatively, high dispersal and competition values (figure 3*e*) allowing coexistence of species and hence high γ-diversity (figure 3*a*) as well as high β-diversity owing to increased spatial turnover across assemblages (figure 3*b*).

Studies suggest that increased competition might promote extinction and speciation simultaneously [17]. This would imply that ‘species diversity itself could be a driver of species diversification’ [79]. Within our models, only MET behaved consistently with an increase in speciation and extinction for strong heterospecific competition (electronic supplementary material, figure S48). However, all our models show that when most suitable sites are reachable for dispersers, higher competition lead to specialists with narrower thermal ranges (ω) and non-overlapping thermal optima ( T ) (electronic supplementary material, figure S44), similar to [80]. Indeed, in ME and MET, populations on partially connected islands (i.e. A and B) exchanged species more frequently, given their dispersal abilities and evolved higher tolerance to other species in order to avoid competition than populations on species-poor and more isolated islands (i.e. C and D) (electronic supplementary material, figure S50). The same was not observed in M0, since competitive traits were modelled to be constant through time.

Higher dispersal abilities are required to maximize allopatric speciation and, thus, biodiversity when competition is high because competition restricts geographic ranges according to landscape structure. Thus, competition modulates connectivity by strongly limiting species geographical ranges, leading to the isolation of populations confined to their home islands (figure 3*a,c,h*). The resulting shift towards narrower thermal widths restrict species range sizes and between-population gene flow, enabling speciation rates to increase and biodiversity to emerge for high dispersal values. This shift of diversity peaks across a dispersal gradient, and its dependency on competition and landscape structure emphasizes the importance of understanding the eco-evolutionary interactions between biotic and abiotic processes [34,81], particularly within a spatio-temporal context [82,83]. Across our landscape, our findings are consistent with regional metacommunity models at shorter temporal scales, where more specialized species coexist when species have high dispersal abilities [84]. Importantly, disturbance may be selected against such highly specialized species, and such disturbances may become particularly apparent over macroevolutionary times (e.g. quaternary sea level changes and temperature oscillations).

Environmental dynamics, e.g. related to temperature and sea level changes, changed connectivity regimes through time and left the signatures of macro-evolutionary processes on biodiversity. Interestingly, increases in extinction events owing to environmental change led to future speciation events when dispersal was low (see M0, for example, in electronic supplementary material, figure S16). This is evident for the within-island speciation events at very low dispersal abilities (i.e. *d* = 0–0.15, figure 3*g*), which only occurred during the more dynamic period (electronic supplementary material, figures S16 and S17) and more frequently at higher competition. This stresses the role of heterospecific competition in isolating local populations through geographical and biotic barriers [4,24]. The importance of geological and environmental dynamics on dispersal, extinction, speciation and the resulting biodiversity has been shown in geologically dynamic systems (e.g. islands or mountains) [85–87]. Environmental dynamics such as habitat fragmentation may accelerate speciation by fostering population isolation, leading to high extinction and fast species turnover [64] while also leading to disruptions in the spread of adaptive traits [25,88]. Fragmentation, climate change and species interactions can also affect trait evolution through selection [89]. For example, an unstable climate may lead to selection for increased dispersal [90], while a stable climate might select for increased competition [91]. With alternative models that allow for dispersal and competitive traits to evolve, we show that dispersal is under much weaker selection than competition (electronic supplementary material, figures S28–S31), especially during stable climates and consistent with theoretical expectations [40,52,92,93]. This suggests that—given a landscape—it may be ‘easier’ for species to ‘evolve’ (e.g. competitive traits) than to ‘move’ (e.g. dispersal ability) [94,95].

Assuming a trade-off between dispersal and competition traits (i.e. the MET model) strengthened the selection of dispersal abilities towards low dispersal (i.e. ‘bad’ dispersers) when competition was high (i.e. simulations with large values of *α*_*fh*_) and environmental dynamics was low. This left a legacy of evolution of dispersal towards lower values in areas with high species richness, since dispersal and competition traits are constrained in the MET model. This arises from stronger heterospecific competition experienced by populations as species accumulate (electronic supplementary material, figure S31). This effect is particularly evident in connectivity regime ϰ1, where species accumulated in islands A and B, while island C contained only a single species throughout the simulations. Such local competition may lead to decreases in thermal ranges (i.e. specialization), increases in tolerance to other species (i.e. heterospecific tolerance) and decreases in dispersal, aligning with the concept of evolutionary pressures towards lower dispersal in areas of high stability and species richness [31,40,84,91]. An unconstrained evolution regarding competition traits (i.e. the ME model) leads to the evolution of physiologically ‘implausible’ species that are extremely tolerant to the presence of other species, increasing γ and α diversity (figure 3*a,c*) and average population sizes (electronic supplementary material, figure S47). Only physiologically implausible species were present at the end of the third dispersal regime for the ME model, since only low tolerance to other species traits was considered plausible under high dispersal, and without constraints, species quickly evolved their competitive traits (figure 2*c*). Competitive ability strongly deviated from a random (non-selective or ‘neutral’) Brownian motion process. It is important to note that the general tendencies found here may be sensitive to the details of the underlying models. Here, we only account for a single trade-off shape and explore a single trait evolutionary rate.

A multitude of interacting processes make process inference challenging, often resulting in under-determination [96] and identifiability [97,98] problems. These relate to challenges in determining the most suitable models to explain a given set of empirical diversity patterns or identifying parameters from empirical data. Even in a single environmental scenario, our simulations revealed complex biodiversity dynamics related to competition and dispersal in full-parameter space exploration. The assumptions of the M0 model are similar to those of many current meta-community models [e.g. 99], but deviate with respect to crucial dispersal mechanisms. While there was no cost of dispersal for M0 and ME, only a simple constrain with competition was tested in MET [52]. Moreover, we did not consider any kind of spatial sorting or more complex forces in favour or against dispersal propensity, such as kin-competition and energetic costs of dispersal [2,31,93,100–102]. Future studies could combine dispersal evolution dynamics at local scale with variable connectivity [31,101,103,104] further linking mechanisms with empirical dispersal evolution data for example [105,106]. Another example is long-distance dispersal which involves rare events driven by complex and highly stochastic processes that change dispersal kernels, such as fecundity propagule density and persistent colonization success [6,7,107,108]. Moreover, the coevolution of many plants with animals, resulting in more intricate dispersal processes such as zoochory and seed predation, were not considered in our models [109]. Thus, alternative costs of dispersal, dispersal kernels, landscape structures and species interaction mechanisms should be explored to assess how general our findings are.

Addressing additional dispersal-related mechanisms could improve our understanding of biodiversity processes that are ideally linked with empirical and measurable quantities [110]. Long-term dispersal, i.e. spanning long periods of time [7], and competition strength, for example, can be estimated from empirical data by understanding landscape and biogeographical histories from phylogenies and fossils, but not without uncertainties [111]. Model modifications may also resemble empirical systems more closely by including traits that act more directly on dispersal, cooccurrence and competition, such as body mass, height, growth rate or more complex biotic interactions (e.g. trophic interactions) [112–114]. Similarly, models could be expanded by considering different trade-off surfaces and mechanisms that include, for example, growth rate as part of the colonization-dispersal trade-off [47,48,50,108,115] or the possibility of sympatric or ecological speciation through trait divergence and/or the consideration of habitat similarity when accounting for Dobzhansky–Muller incompatibilities [116].

In summary, we stress the nonlinear interactions between dispersal, competition and landscape structure in shaping biodiversity. We highlight the pivotal role of dispersal in modulating the emergence of biodiversity according to competition and landscape structure and how these leave discernible marks on biodiversity in a macroecological context. We emphasize the importance of ecological and evolutionary drivers within known landscape histories for our understanding of the mechanisms driving biodiversity. This intricate interplay between ecological interactions and evolutionary processes underscores the need for further research that integrates both spatial and deep-time dynamics to fully grasp the mechanisms driving biodiversity.

## Data Availability

The simulations and analyses are entirely reproducible. The scripts, required files, supplementary materials, and animations can be accessed at [117] and permanent at [118]. Electronic supplementary material is available online [119].
